# Favorable outcome of *NUTM1*-rearranged infant and pediatric B cell precursor acute lymphoblastic leukemia in a collaborative international study

**DOI:** 10.1038/s41375-021-01333-y

**Published:** 2021-07-01

**Authors:** Judith M. Boer, Maria Grazia Valsecchi, Femke M. Hormann, Željko Antić, Marketa Zaliova, Claire Schwab, Giovanni Cazzaniga, Chloé Arfeuille, Hélène Cavé, Andishe Attarbaschi, Sabine Strehl, Gabriele Escherich, Toshihiko Imamura, Kentaro Ohki, Tanja A. Grüber, Rosemary Sutton, Agata Pastorczak, Tim Lammens, Frédéric Lambert, Chi Kong Li, Enrique Carrillo de Santa Pau, Steve Hoffmann, Anja Möricke, Christine J. Harrison, Monique L. Den Boer, Paola De Lorenzo, Ronald W. Stam, Anke K. Bergmann, Rob Pieters

**Affiliations:** 1 Princess Máxima Center for Pediatric Oncology, Utrecht, Netherlands; 2 Oncode Institute, Utrecht, Netherlands; 3 Interfant Trial Data Center, School of Medicine and Surgery, University of Milan-Bicocca, Monza, Italy; 4 Institute of Human Genetics, Medical School Hannover, Hannover, Germany; 5 CLIP, Department of Pediatric Hematology and Oncology, Second Faculty of Medicine, Charles University and University Hospital Motol, Prague, Czech Republic; 6 Leukaemia Research Cytogenetics Group, Wolfson Childhood Cancer Research Centre, Translational and Clinical Research Institute, Newcastle University, Newcastle upon Tyne, United Kingdom; 7 Tettamanti Research Center, Pediatric Clinic, University of Milan-Bicocca, Monza, Italy; 8 Department of Genetics, Robert Debré Hospital and University of Paris, Paris, France; 9 Department of Pediatric Hematology and Oncology, St. Anna Children’s Hospital, Medical University of Vienna, Vienna, Austria; 10 CCRI, St. Anna Children’s Cancer Research Institute, Vienna, Austria; 11 Department of Pediatric Hematology and Oncology, University Medical Center Hamburg, Hamburg, Germany; 12 Department of Pediatrics, Kyoto Prefectural University of Medicine, Kyoto, Japan; 13 Department of Pediatric Hematology and Oncology Research, National Research Institute for Child Health and Development, Tokyo, Japan; 14 Department of Oncology, St. Jude Children’s Research Hospital, Memphis, TN USA; 15 Children’s Cancer Institute Australia, Lowy Cancer Research Centre, University of NSW, Randwick, NSW Australia; 16 Department of Pediatric Oncology and Hematology, Medical University of Lodz, Lodz, CA Poland; 17 Cancer Research Institute Ghent (CRIG), Ghent, Belgium; 18 Department of Pediatric Hemato-oncology and Stem Cell Transplantation, Ghent University Hospital, Ghent, Belgium; 19 Centre Hospitalier Universitaire de Liège, Liege, Belgium; 20 Department of Paediatrics, The Chinese University of Hong Kong, Shatin, Hong Kong; 21 Computational Biology Group, Precision Nutrition and Cancer Research Program, IMDEA Food Institute, Madrid, Spain; 22 Computational Biology, Leibniz Institute on Ageing-Fritz Lipmann Institute (FLI), Jena, Germany; 23 Department of Pediatrics, Christian-Albrechts-University Kiel and University Medical Center Schleswig-Holstein, Kiel, Germany; 24 Erasmus MC-Sophia Children’s Hospital, Department of Pediatric Oncology/Hematology, Rotterdam, Netherlands

**Keywords:** Acute lymphocytic leukaemia, Genetic translocation

## To the Editor:

Fusions of *NUTM1* (15q14) define a novel subtype of B-cell precursor acute lymphoblastic leukemia (B-ALL) that is mutually exclusive with sentinel leukemia-driving aberrations based on RNA-sequencing

in children [1–5] and infants [3, 6]. *NUTM1* fusions are characterized by increased expression of almost the complete open reading frame of *NUTM1* fused to one of several fusion partners. Downstream effects of *NUTM1* fusions include upregulation of the proto-oncogene *BMI1* [3]. In addition, specific *NUTM1* fusions are associated with *HOXA* gene cluster upregulation [1, 3]. The incidence of *NUTM1*-rearranged ALL appears to be low, but reported series are small. The subtype may be more prevalent in infants without *KMT2A* rearrangement, suggested by RNA-sequencing [3, 6] and 15q aberrations [7]. Since not all *NUTM1* fusions are obvious from the karyotype [8], the estimated frequency could be higher. We determined the frequency, characteristics, and outcome of *NUTM1*-rearranged B-ALL in a large series of 85 cases in a Ponte di Legno Childhood ALL Working Group study.

Interfant-related study groups provided *NUTM1* screening results for *KMT2A*-wildtype infants from the Interfant-99 [9] and -06 [10] cohorts (2000–2016) with a karyotypic 15q aberration, a normal karyotype, or missing karyotype (Supplementary Fig. S1). Separately, *NUTM1*-rearranged cases of any age, diagnosed between 1986 and 2019, were collected from the Ponte di Legno consortium (Supplementary Table S1). In accordance with the declaration of Helsinki, written informed consent was obtained from parents or guardians, and institutional review boards approved the use of excess diagnostic material for research purposes. Techniques used for the detection of *NUTM1* rearrangement are described in Supplementary Methods. Categorical variables were compared by Fisher’s exact test, continuous variables by Wilcoxon rank-sum test with continuity correction. Survival was estimated according to Kaplan–Meier and standard error according to Greenwood. Curves were compared by log-rank test. Cox proportional hazard regression was used to estimate the impact of *NUTM1* rearrangement in a multivariate model. All tests were two-sided. Analyses were performed using SAS 9.4 and R version 3.2.2.

Among 161 evaluated *KMT2A*-wildtype Interfant-enrolled infants with B-ALL, 35 (21.7%) were *NUTM1*-rearranged (Supplementary Fig. S1 and Supplementary Table S2). The tested cases did not differ in baseline characteristics from the 73 untested Interfant cases (Supplementary Table S3). We also collected *NUTM1*-rearranged cases of all ages in the Ponte di Legno consortium, resulting in 11 infants and 32 children ≥1 year. Based on three population-representative cohorts, the frequency of *NUTM1* rearrangement in pediatric B-ALL ranged from 0.28 to 0.86% (Supplementary Tables S2 and S4). We extended the cohort with seven pediatric cases for which baseline and molecular features were published [2] to a total of 85 *NUTM1*-rearranged cases (Supplementary Fig. S2 and Supplementary Table S5). The median age in infants was 5.6 months (range 0.43–11.0 months) and in children 4 years (range 1–15 years). White blood cell counts (WBC) were more often ≥50x10e-9/L in infants (45%) than in children (13%, *p* = 0.002). Among children, a nonsignificant skewing towards male patients was observed (74%), while the gender ratio in infants was balanced (54% male; *p* = 0.072). Deletions of *IKZF1*, *PAX5*, *ETV6*, or *CDKN2A/B* were rare in *NUTM1*-rearranged cases: at least one of these genes was deleted in only 4/38 cases analyzed (Supplementary Table S6). For 32 *NUTM1*-rearranged infants with immunophenotype data, 16 were pre-B, 12 common, 3 pro-B ALL, and one biphenotypic acute leukemia. Remarkably, all three pro-B ALL cases had a *BRD9-NUTM1* fusion. Among *KMT2A*-wildtype infant ALL, 54% of *NUTM1*-rearranged were <6 months of age at diagnosis compared with 16% of the *NUTM1*-wildtype group (*p* < 0.0001). Other baseline characteristics were similar between *NUTM1*-rearranged and *NUTM1*-wildtype*/KMT2A*-wildtype infants (Supplementary Table S7).

**Fig. 1:**
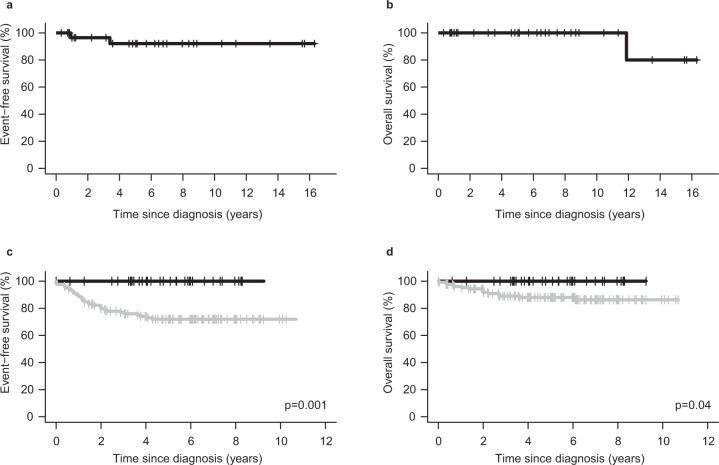
Outcome of *NUTM1*-rearranged ALL. Event-free (**a**) and overall (**b**) survival in children ≥1 year with *NUTM1* rearrangement collected in the Ponte di Legno cohort (*N* = 32). Event-free (**c**) and overall (**d**) survival by *NUTM1* status in Interfant protocols. Black curve, *NUTM1*-rearranged (*N* = 35); grey curve, *NUTM1*-wildtype*/KMT2A*-wildtype (*N* = 126). For the event table of Interfant cases see Supplementary Table S8.

All *NUTM1*-rearranged cases reached complete remission. Moreover, 41 of 45 (91.1%) had minimal residual disease <0.05%, which is favorable compared with, for example, 79.3% in childhood ALL cases in the DCOG-ALL10 cohort [11] and 48% in *KMT2A*-rearranged infant ALL [12]. No *NUTM1*-rearranged patient received stem cell transplant in first remission (Supplementary Table S6). We studied long-term outcomes separately for three groups: children ≥1 year and infant cases <1 year collected in the Ponte di Legno cohort, and infants treated with the Interfant protocols. Among 32 children ≥1 year (median follow-up 6.0 years), one isolated bone marrow and one isolated CNS relapse occurred. Both patients reached the second remission; the child with CNS relapse died in remission 10 years later leaving 31/32 alive at the last follow-up. This resulted in a 4-year event-free and overall survival of 92.1% (95% CI 82.0–100) and 100%, respectively for childhood *NUTM1*-rearranged ALL (Fig. 1a, b). These results, obtained from cases enrolled on different treatment protocols over more than two decades, suggest that *NUTM1* is a favorable childhood ALL subtype. Among 11 infants (median follow-up 1.7 years) from the Ponte di Legno cohort one isolated CNS relapse was reported; this patient reached the second remission and underwent SCT. For uniformly treated infants in the Interfant protocols, we compared outcomes between 35 *NUTM1*-rearranged and 126 *NUTM1*-wildtype*/KMT2A*-wildtype cases. The 4-year event-free survival was 100 versus 74.0% (95% CI 65.1–81.0), respectively (*p* = 0.001). The better outcome of *NUTM1*-rearranged infants compared with *NUTM1*-negative ones was confirmed after adjusting by WBC, gender, and prednisone response (*p* = 0.0001). The 4-year overall survival was 100% in *NUTM1*-rearranged cases versus 88.0% (95% CI 80.5–92.7; *p* = 0.04) in *NUTM1*-wildtype*/KMT2A*-wildtype cases (Fig. 1c, d and Supplementary Table S8).

**Fig. 2:**
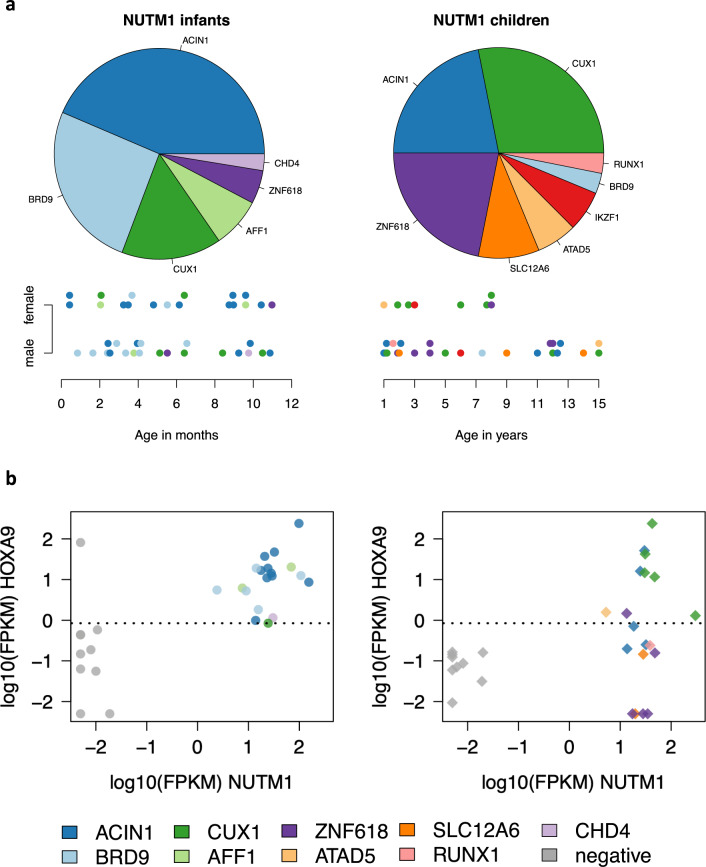
Different *NUTM1* partners in infants and children. **a** Pie diagrams with the proportions of different *NUTM1* partner genes among infants (*N* = 39; left) and children from the age of 1 (*N* = 32; right) with a known fusion partner. Below the pie diagrams, each patient is represented by a dot color-coded for the fusion, depicted along age at diagnosis (x-axis) for infants (in months; left) and children (in years; right) and gender (y-axis). Dots are plotted with a small offset to avoid overlap. **b** Dot plot showing *NUTM1* and *HOXA9* expression. Infant cases (*N* = 19; left panel) are indicated by a circle, pediatric cases (*N* = 19; right panel) by a diamond. Ten infants and nine children with B-ALL without *NUTM1* or *KMT2A* rearrangement were included as controls (gray). Counts from RNA-sequencing libraries were normalized to fragments per kilobase per million (FPKM), 0 was substituted by 0.005, subsequently FPKM values were log10-transformed. The median *HOXA9* expression across all 57 samples was 0.85 FPKM (log10 value–0.073), indicated by the dotted line.

Ten fusion partners of *NUTM1* were identified among 71 cases with known partners. Among infants *ACIN1* (17 cases, 44%), *BRD9* (ten cases, 26%), and *CUX1* (six cases, 15%) were most frequently observed, and among children *CUX1* (nine cases, 28%), *ZNF618* (nine cases, 28%), and *ACIN1* (seven cases, 22%) (Fig. 2a and Supplementary Table S9). Interestingly, recurrent fusions found among infants belong to a previously described set of partner genes associated with upregulation of HOXA cluster genes: *ACIN1*, *CUX1*, AFF1, and *ZNF618* [1, 3]. We confirmed *HOXA9* expression in *NUTM1*-rearranged cases with *ACIN1*, *CUX1*, and *AFF1*, and additionally found that *BRD9-NUTM1* was associated with *HOXA9* expression. In contrast, *HOXA9* expression was low or absent in 4/5 pediatric *ZNF618-NUTM1* fusion cases (Fig. 2b). The only infant with this fusion was 11 months old, therefore this fusion could biologically be more similar to pediatric-type fusions. We found one recently described and two novel *NUTM1* fusion partners: *ATAD5* (two pediatric cases) [4], *CHD4* (infant, 9 months), and *RUNX1* (pediatric case). Cases with one of these fusions showed *HOXA9* expression around the median (Fig. 2b).

To further unravel mechanisms driving dysregulation of the HOXA gene cluster, we obtained data sets generated through the BLUEPRINT project [13] (Supplementary Table S10). RNA-sequencing showed high expression of genes in the HOXA locus, particularly of *HOXA9* and *HOXA10*, in the *ACIN1-NUTM1* case, comparable with levels observed in two cases with *KMT2A* rearrangement (Supplementary Fig. S3). Bisulfite sequencing and chromatin immunoprecipitation sequencing revealed decreased methylation levels around *HOXA9* and *HOXA10*, accompanied by high H3K4me3 and H3K27Ac signals (chromatin state E11), which is indicative of the presence of an active transcription start site. Furthermore, in contrast to the other ALL subtypes, the HOXA region including *HOXA9* and *HOXA10* in the *ACIN1-NUTM1* case was characterized by an alternating pattern of activating (H3K4me1 and H3K4me3) and repressing (H3K27me3) histone modifications. While the locus appeared to be constitutively targeted by the Polycomb complex (chromatin state E7), two short, activated regions overlapping with *HOXA9* escape the repression. Together, these data suggest that the *ACIN1-NUTM1* fusion protein drives upregulation of the HOXA gene cluster, particularly the *HOXA9* and *HOXA10* genes, and that this process is associated with epigenetic modifications of CpGs and chromatin.

In the present study, we have shown that, like *KMT2A* rearrangement, *NUTM1* rearrangement is more frequent in infants (around 3–5%) than in children (0.4–0.9%). In contrast to *KMT2A* rearrangement, which is found in ~10% of adult B-ALL, *NUTM1* fusions have at least so far not been reported in adults [1, 2](own unpublished observation). While in *KMT2A*-rearranged infant ALL, age at diagnosis <6 months is associated with higher relapse risk, *NUTM1*-rearranged infants tended to be younger than *NUTM1*-wildtype*/KMT2A*-wildtype infants. Most striking is the excellent outcome of *NUTM1*-rearranged infant and childhood ALL.

We postulate that there are two biological subgroups within the *NUTM1* subtype. Firstly, a *HOXA9*-positive *NUTM1* subgroup involving a limited number of partners (*ACIN1*, *CUX1*, *BRD9*, and *AFF1*) prevalent among infants <9 months, and secondly, a *HOXA9*-negative *NUTM1* subgroup, emerging in infants close to 1 year old and increasing to almost half of *NUTM1*-rearranged pediatric cases. We previously showed that both types of fusions upregulated genes on 10p12.31-12.2, including *BMI1* [3], which may be regulated via binding of the histone acetyltransferase EP300 to *NUTM1* [14]. HOXA-upregulating fusions might directly bind to gene promoters in the HOXA cluster via a DNA binding domain from the partner gene and affect local DNA methylation, histone modification, and gene expression. HOXA upregulation has recently been associated with sensitivity to inhibitors of the KMT2A-Menin complex in *KMT2A*-rearranged acute leukemia; it would be interesting to assess whether HOXA-upregulating *NUMT1*-rearranged ALL cells are similarly sensitive.

Since new *NUTM1* partners are still being discovered, an unbiased detection approach is preferred. *NUTM1* rearrangements can be detected at diagnosis using either break-apart FISH or, preferably, using RNA-sequencing. In addition, the finding that both RNA expression of the 3′ exons [3] and protein expression [8] are highly specific for fusion cases could aid in their detection. The favorable outcome of *NUTM1*-rearranged cases, mostly treated on standard risk arms, might allow treatment reduction similar to that applied for *ETV6-RUNX1* [11, 15]. Further delineation of genetic subtypes in *KMT2A*-wildtype infant ALL may argue for a redefinition of “infant ALL”, and thereby Interfant inclusion criteria. In conclusion, this collaborative international study characterizes *NUTM1*-rearranged infant and pediatric B-ALL as a very good prognostic subtype.

## Supplementary information

Supplemental Material

Supplemental Table 5
