# Supplementary material for: Favorable outcome of NUTM1-rearranged infant and pediatric B cell precursor acute lymphoblastic leukemia in a collaborative international study
Source: Leukemia. 2021 Jul 1;35(10):2978–82. doi: 10.1038/s41375-021-01333-y (PMC8478641; doi:10.1038/s41375-021-01333-y)
Supplement: Supplementary file 1 — Supplemental Material [file 41375_2021_1333_MOESM1_ESM.pdf]

Supplementary Material for

Favorable outcome of *NUTM1*-rearranged infant and pediatric B cell precursor acute lymphoblastic leukemia in a collaborative international study

Boer J.M. et al.

SUPPLEMENTARY METHODS

Detection of *NUTM1* rearrangement

SUPPLEMENTARY TABLES

Supplementary Table S1. Number of *NUTM1*-rearranged cases (any age) contributed by each study group to the Ponte di Legno cohort

Supplementary Table S2. Frequency of *NUTM1* rearrangement in infant and pediatric B-ALL cohorts

Supplementary Table S3. Comparison of Interfant *KMT2A*-wildtype B-ALL cases tested and untested for *NUTM1* rearrangement

Supplementary Table S4. Comparison of tested and untested UKALL B-ALL cases

Supplementary Table S5. *NUTM1*-rearranged cases: separate Excel table

Supplementary Table S6. Clinical and molecular characteristics of *NUTM1*-rearranged ALL in infants and children

Supplementary Table S7. Comparison of Interfant *NUTM1*-rearranged cases versus *NUTM1*-wildtype/*KMT2A*-wildtype infant B-ALL cases

Supplementary Table S8. Events in survival analysis of Interfant study cohort

Supplementary Table S9. Frequency of the different *NUTM1* partner genes

Supplementary Table S10. Individual datasets used in this study obtained from Blueprint project

SUPPLEMENTARY FIGURES

Supplementary Figure S1. Consort diagram of Interfant-99/06 cohort screen

Supplementary Figure S2. Diagram overview of study cases

Supplementary Figure S3. HOXA cluster epigenetic landscape

SUPPLEMENTARY REFERENCES

SUPPLEMENTARY FIGURE LEGENDS

SUPPLEMENTARY METHODS

Detection of *NUTM1* rearrangement

Techniques used for the detection of *NUTM1* rearrangement were break-apart FISH (Cytocell probe set MPH4800; ZytoVision ZytoLight ® SPEC NUTM1 Dual Color Break Apart Probe Z-2208-200; Abnova NUTM1 Split (FS0030); or NUT/BRD4(19p13.1), Fa Empire Genomics), targeted or transcriptome RNA-sequencing, RT-PCR and Sanger sequencing for known fusions or 3' expression of *NUTM1* using exon 6 forward primer (5'- TGG-GGA-GTC-AGA-TGG-AAA-ACA -3') and exon 8 reverse primer (5'- CCC-ATC-CCC-ATC-TTC-ATC-CT-3'), and karyotyping.[1-5]

SUPPLEMENTARY TABLES

Supplementary Table S1. Number of *NUTM1*-rearranged cases (any age) contributed by each study group to the Ponte di Legno cohort

**Study group**	**Total**	**Infants***	**Children**
Polish Pediatric Leukaemia and Lymphoma Study Group (PPLLSG)	2	1	1
Berlin-Frankfurt-Münster – Austria (BFM-A)	5		5
Berlin-Frankfurt-Münster - Germany/Switzerland (BFM-G/CH)	7	1	6
Childhood Leukaemia Investigation Prague (CLIP)	4	2	2
Cooperative Study Group for Childhood ALL (COALL)	2	1	1
Dutch Childhood Oncology Group (DCOG)	4		4
Japanese Association of Childhood Leukaemia Study (JACLS)	3		3
National Cancer Research Institute Childhood Cancer and Leukaemia Group Leukaemia Subgroup (NCRI CCLG LSG)	6		6
St Jude's Children's Research Hospital (SJCRH)	4	2	2
Tokyo Children's Cancer Research Group (TCCSG)	1		1
Société Française de la lutte contre les Cancers et les leucémies de l'Enfant et de l'Adolescent (SFCE)	5	4	1
Gu et al. Nature Genetics 2019	7		7
**Total**	**50**	**11**	**39**

***** Not enrolled on Interfant-99/06 study

Supplementary Table S2. Frequency of *NUTM1* rearrangement in infant and pediatric B-ALL cohorts

**Cohort**	**Age (years)**	**Screening method**	**B-ALL screened / B-ALL total (%)**	**B-ALL screened / *KMT2A*-wildtype B-ALL (%)**	***NUTM1*-rearranged / B-ALL screened (%)**	***NUTM1*-rearranged / B-ALL total (%)**	**Reference**
Interfant99[a]/06[b] B-ALL[c]	<1	Several	161/1108 (14.5%)	161/234 (68.8%)	35/161 (21.7%)	35/1108 (3.2%)	Pieters et al. 2007, 2019, and current study
CLIP[d]	1-18	RNA-seq	146/579 (25.2%)	-	2/146 (1.4%)	2/579 (0.35%)	Current study
DCOG-ALL8/9/10 COALL97/03[e]	1-18	Expression array	210/583 (36.0%)	-	5/210 (2.4%)	5/583 (0.86%)	Hormann et al. 2019
UKALL2003[f]	1-24	FISH	216/796 (27.1%)	-	2/216 (0.93%)	2/796 (0.28%)	Current study

[a]Interfant-99: 464 non-T-ALL cases of 482 total enrolled (Pieters et al. 2007)

[b]Interfant-06: 644 non-T-ALL cases of 651 total enrolled (Pieters et al. 2019)

[c]Interfant-99/06: screened cases were representative for the complete cohort of *KMT2A*-wildtype B-ALL (**Supplementary Table S3**)

[d]CLIP: consecutively diagnosed BCP-ALL between December 2010 - April 2020, screened cases were negative for high hyperdiploidy (> 50 chr), *ETV6-RUNX1*, hypodiploidy (≤ 44 chr), *KMT2A* rearrangement, *TCF3-PBX1, BCR-ABL1*

[e]DCOG/COALL: population-based cohort (van der Veer et al. Blood 2013), screened cases were negative for high hyperdiploidy (> 50 chr), *ETV6-RUNX1*, hypodiploidy (≤ 44 chr), *KMT2A* rearrangement, *TCF3-PBX1, BCR-ABL1*

[f]UKALL2003: population-based cohort, screened cases were negative for high hyperdiploidy (> 50 chr), *ETV6-RUNX1*, hypodiploidy (≤ 44 chr), *KMT2A* rearrangement, *TCF3-PBX1, BCR-ABL1*, iAMP21 and *TCF3-HLF*; small bias towards screening of younger cases and higher white blood cell count (**Supplementary Table S4**)

Supplementary Table S3. Comparison of Interfant *KMT2A*-wildtype B-ALL cases tested and untested for *NUTM1* rearrangement

Characteristics	Total N=234	Tested N=161	Untested N=73	Tested vs. Untested P-value[a]
**Age in months**<br>median<br>range	 8.7<br>0.07-12.0	 8.7<br>0.07-11.9	 8.7<br>1.9-12.0	0.86
Age <6 months	55/234 (24%)	39/161 (24%)	16/73 (22%)	0.74
WBC ≥300x10^9/L [b]	13/233 (6%)	10/160 (6%)	3/73 (4%)	0.76
Gender male	134/234 (57%)	90/161 (56%)	44/73 (60%)	0.57
Prednisone poor response[c]	34/225 (15%)	19/155 (12%)	15/70 (21%)	0.11
No CR after induction	3/234 (1.3%)	2/161 (1.2%)	1/73 (1.4%)	1
MRD TP1[d] ≥0.05%	16/84 (19%)	13/65 (20%)	3/19 (16%)	0.99
Event-free survival at 4 years (95% CI)	77.3% (71.2 – 82.3)	79.7% (72.3 – 85.3)	72.3% (60.4 – 81.2)	0.25
Overall survival at 4 years (95% CI)	88.4% (83.4 – 92.0)	90.6% (84.7 – 94.4)	83.5% (72.8 – 90.3)	0.12

Out of 234 patients, 158 (68%) were treated with Interfant-06 and 76 (32%) with Interfant-99. The proportion of tested patient did not differ by protocol given: 106 (67%) and 55 (72%) with Interfant-06 and Interfant-99, respectively (p=0.45). CR, complete remission. WBC, white blood cell count.

[a] Categorical variables were tested by Fisher's exact test, continuous variables by Wilcoxon rank sum test with continuity correction. Survival curves were compared by log-rank test.

[b] White blood cell count was unknown in 1 tested case

[c] Prednisone response data were available for 225 cases overall (155 tested and 70 untested cases)

[d] Minimal residual disease after the first induction course of chemotherapy (PCR and/or FCM)

Supplementary Table S4. Comparison of tested and untested UKALL B-ALL cases

		NUTM1		
		**Not tested**	**Tested**	**p-value***
**Total (%)**		580 (72.9)	216 (27.1)	
**Age**	**1-4**	205 (35.3)	105 (48.6)	
	**5-9**	142 (24.5)	36 (16.7)	
	**10-15**	153 (26.4)	51 (23.6)	
	**16+**	80 (13.8)	24 (11.1)	0.005
**Regimen**	**A**	236 (40.7)	84 (38.9)	
	**B**	204 (35.2)	81 (37.5)	
	**C**	140 (24.1)	51 (23.6)	0.826
**Sex**	**Female**	250 (42.6)	92 (43.1)	
	**Male**	330 (57.4)	124 (56.9)	0.897
**WBC**	**<50**	482 (83.1)	159 (73.6)	
	**>50**	98 (16.9)	57 (26.4)	0.003

*p-values testing for proportion equivalence in tested cohort verses overall (n=796) cohort

Supplementary Table S5. *NUTM1*-rearranged cases

See separate Excel table

Supplementary Table S6. Clinical and molecular characteristics of *NUTM1*-rearranged ALL in infants and children

Characteristics	Total N=85	Infants N=46	Children N=39	Fisher P Infants vs. Children
**Age in years**<br>median<br>range	 0.88<br>0.04-15	 0.46<br>0.04-0.92	 4<br>1-15	-
Age ≥10 years			10/39 (25.6%)	-
WBC ≥50x10^9/L	23/76 (30%)	20/44 (45%)	5/38 (13%)	0.002
Gender male	54/85 (64%)	25/46 (54%)	29/39 (74%)	0.07
NCI high risk[a]			14/38 (37%)	-
Prednisone poor response[b]	5/60 (8%)	2/38 (6%)	3/22 (14%)	0.35
No CR after induction[d]	0/78 (0%)	0/46 (0%)	0/32 (0%)	-
MRD TP1[c] ≥0.05%	4/45 (9%)	3/25 (12%)	1/20 (5%)	0.62
IKZF1 deletion	2/37 (5%)	0/10 (0%)	2/27 (7%)	-
PAX5 deletion	0/38 (0%)	0/10 (0%)	0/28 (0%)	-
CDKN2A/B deletion	2/38 (5%)	0/10 (0%)	2/28 (7%)	-
ETV6 deletion	2/38 (5%)	0/10 (0%)	2/28 (7%)	-

[a]NCI-Rome high risk defined in children by white blood cell count (WBC) ≥50x10^9/L and/or age ≥10 years

[b]Prednisone response on day 8 ≥1.0x10^9 blast/L

[c]Minimal residual disease after the first induction course of chemotherapy (PCR and/or FCM)

[d]Complete remission (CR) after induction is defined on morphological grounds by the presence of <5% leukemic blasts and regenerating hematopoiesis

Supplementary Table S7. Comparison of Interfant *NUTM1*-rearranged cases versus *NUTM1*-wildtype/*KMT2A*-wildtype infant B-ALL cases

**Characteristics**	**Total N=161**	***NUTM1*-rearranged N=35**	***NUTM1*-wildtype N=126**	***NUTM1*-rearranged vs. wildtype P-value**[a]
**Age in months**<br>median<br>range	 8.8<br>0.07-11.9	 5.6<br>0.43-11.0	 9.3<br>0.07-11.9	<0.00001
Age <6 months	39/161 (24%)	19/35 (54%)	20/126 (16%)	<0.0001
WBC ≥300x10^9/L [b]	10/160 (6%)	2/35 (6%)	8/125 (6%)	1
Gender male	90/161 (56%)	18/35 (51%)	72/126 (57%)	0.57
Prednisone poor response[c]	19/155 (12%)	2/33 (6%)	17/122 (14%)	0.37
No CR after induction	2/161 (1.2%)	0/35 (0%)	2/126 (1.6%)	1
MRD TP1[d] ≥0.05%	13/65 (20%)	2/15 (13%)	11/50 (22%)	0.71
Event-free survival at 4 years (95% CI)	79.7% (72.3 – 85.3)	100%	74.0% (65.1-81.0)	0.001
Overall survival at 4 years (95% CI)	90.6% (84.7 – 94.4)	100%	88.0% (80.5-92.7)	0.04

Out of 161 tested patients, 106 (66%) were treated with Interfant-06 and 55 (33%) with Interfant-99. The proportion of *NUTM1*-rearranged patients did not differ by protocol given: 26 (24.5%) and 9 (16.4%) with Interfant-06 and Interfant-99, respectively (p=0.31). CR, complete remission. WBC, white blood cell count.

[a] Categorical variables were tested by Fisher's exact test, continuous variables by Wilcoxon rank sum test with continuity correction. Survival curves were compared by log-rank test.

[b] White blood cell count was not available in 1 *NUTM1* wildtype case

[c] Prednisone response data available for 33 *NUTM1*-rearranged and 122 *NUTM1* wildtype cases

[d] Minimal residual disease after the first induction course of chemotherapy (PCR and/or FCM)

Supplementary Table S8. Events in survival analysis of Interfant study cohort

	***NUTM1*-rearranged** N=35	***NUTM1*-wildtype** N=126	**Total** N=161
**Events**	0	33	33
**Death in induction**	0	1	1
**Resistance (deaths)**	0	2	2 (0)
**Relapse (deaths)**	0	27	27 (11)
**Deaths in CCR**	0	3	3
**Alive in CCR**	35	93	128

Supplementary Table S9. Frequency of the different *NUTM1* partner genes

	Total		Infant		Child	
**Partner**	N	%	N	%	N	%
ACIN1 (14q11.2)	24	33.8	17	43.6	7	21.9
AFF1 (4q21.3-q22.1)	3	4.2	3	7.7	0	0
ATAD5 (17q11.2)	2	2.8	0	0.0	2	6.3
BRD9 (5p15.33)	11	15.5	10	25.6	1	3.1
CHD4 (12p13.31)	1	1.4	1	2.6	0	0
CUX1 (7q22.1)	15	21.1	6	15.4	9	28.1
IKZF1 (7p12.2)	2	2.8	0	0	2	6.3
RUNX1 (21p22.12)	1	1.4	0	0	1	3.1
SLC12A6 (15q14)	3	4.2	0	0	3	9.4
ZNF618 (9q32)	9	12.7	2	5.1	7	21.9
Total with known partner	71	100	39	100	32	100
Unknown partner*	14		7		7	
Grand total	85		46		39	

*Involvement of *NUTM1* confirmed by FISH only or by expression of exons 6-8 of *NUTM1* by RT-PCR

Supplementary Table S10. Individual datasets used in this study obtained from Blueprint project

ID	Subtype	BS-Seq ID	WGBS data allocation	ChIP-Seq ID	RNA-Seq ID
S01GTB	*ACIN1-NUTM1*	S01GTBA1	EGAD00001002313	S01GTBH1	S01GTB11
S017D5	*KMT2A-AFF1*	S017D5A1	Not publicly available	S017D5H1	S017D511
S017B9	*KMT2A-AFF1*	S017B9A1	EGAD00001002313	S017B9H1	S017B911
S01GRF	*TCF3-PBX1*	S01GRFA1	EGAD00001002313	S01GRFH1	S01GRF11
S0179D	*ETV6-RUNX1*	S0179DA1	EGAD00001002313	S0179DH1	S0179D11

SUPPLEMENTARY FIGURES

Supplementary Figure S1. Consort diagram of Interfant-99/06 cohort screen

Of tested *KMT2A*-wildtype infant B-ALL:
35/161=21.7% *NUTM1*-rearranged

Of tested non-sentinel infant B-ALL:
35/136=25.7% *NUTM1*-rearranged

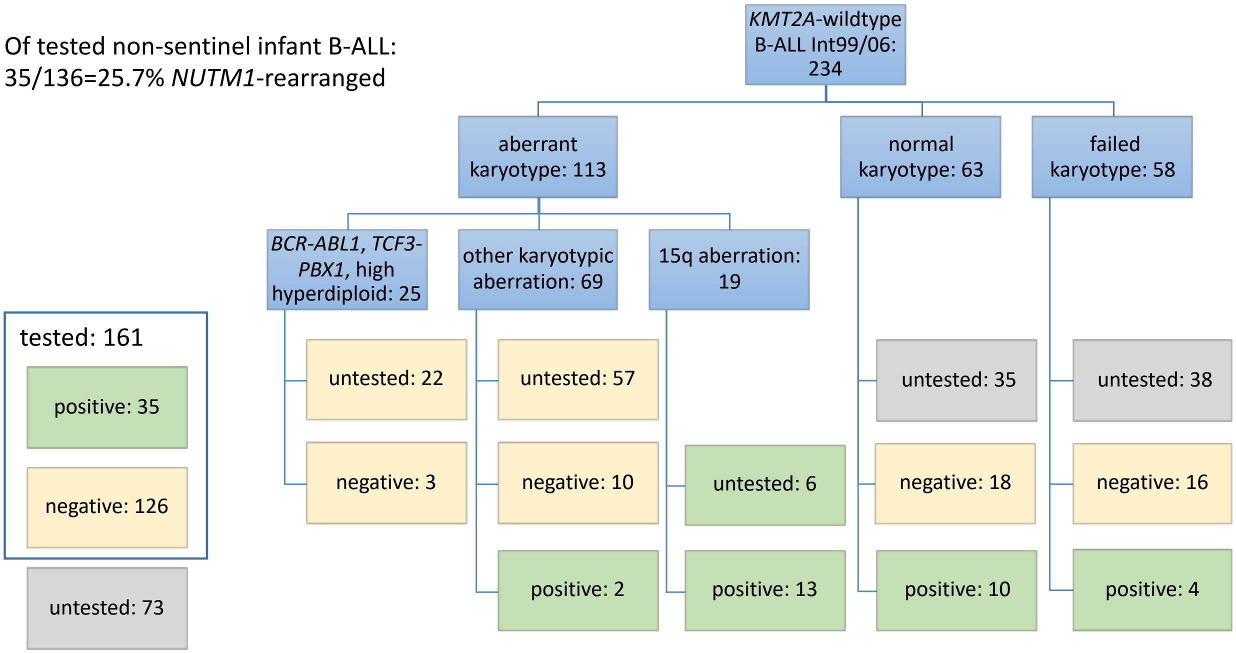

Supplementary Figure S2. Diagram overview of study cases

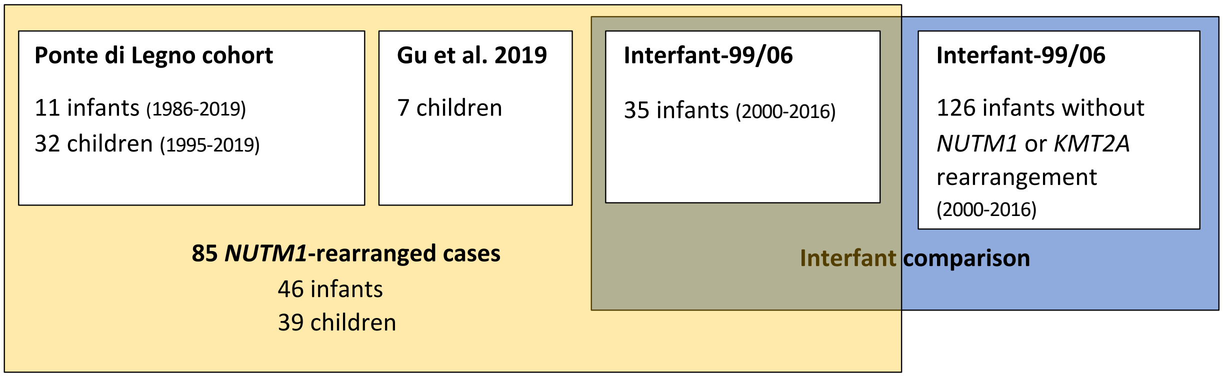

Supplementary Figure S3. HOXA cluster epigenetic landscape

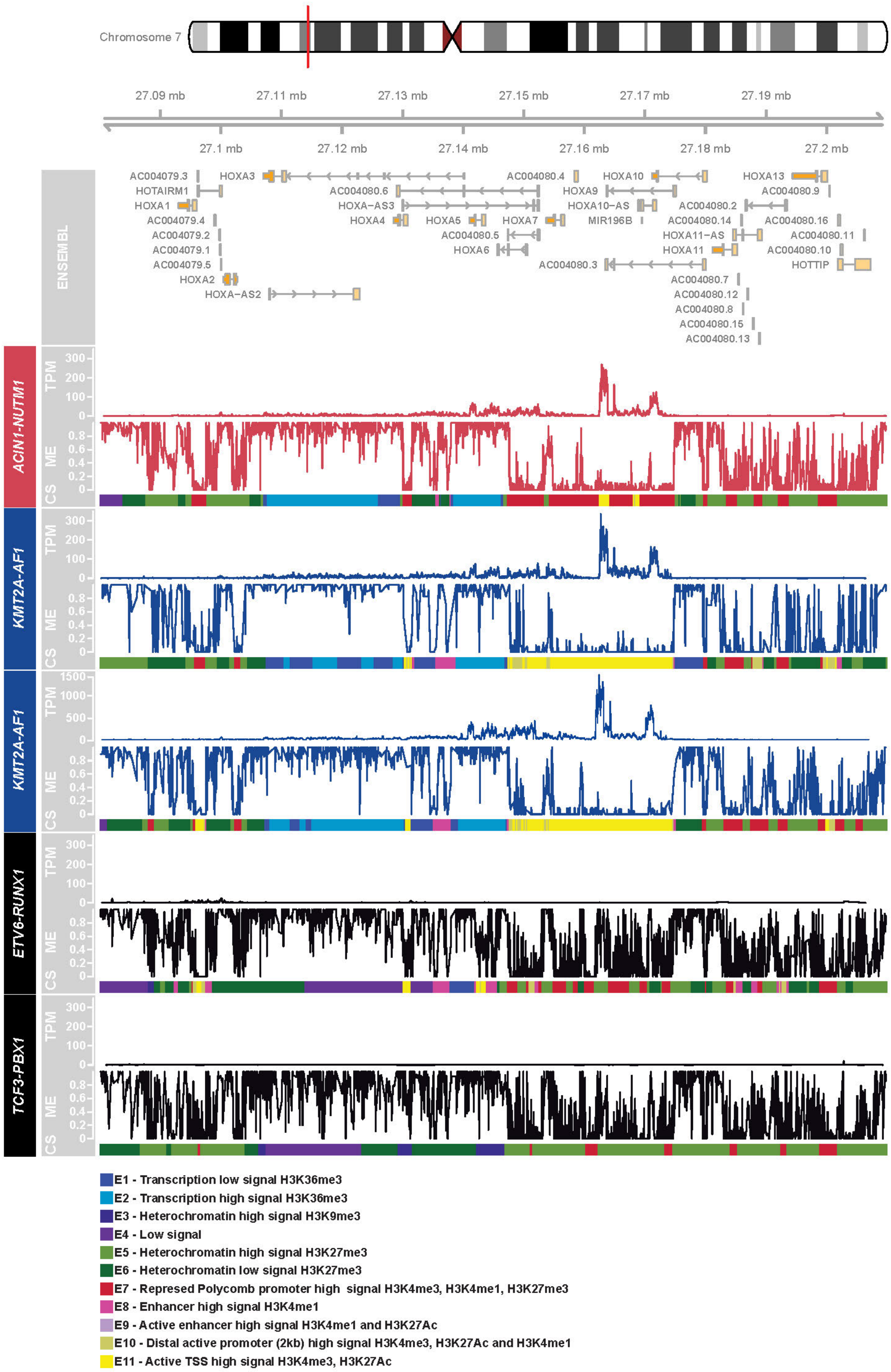

SUPPLEMENTARY REFERENCES

1. Li JF, Dai YT, Lilljebjorn H, Shen SH, Cui BW, Bai L*, et al.* Transcriptional landscape of B cell precursor acute lymphoblastic leukemia based on an international study of 1,223 cases. *Proceedings of the National Academy of Sciences of the United States of America* 2018 Dec 11; **115**(50)**:** E11711-E11720.

2. Gu Z, Churchman ML, Roberts KG, Moore I, Zhou X, Nakitandwe J*, et al.* PAX5-driven subtypes of B-progenitor acute lymphoblastic leukemia. *Nat Genet* 2019 Feb; **51**(2)**:** 296-307.

3. Andersson AK, Ma J, Wang J, Chen X, Gedman AL, Dang J*, et al.* The landscape of somatic mutations in infant MLL-rearranged acute lymphoblastic leukemias. *Nature Genetics* 2015 Apr; **47**(4)**:** 330-337.

4. Gu Z, Churchman M, Roberts K, Li Y, Liu Y, Harvey RC*, et al.* Genomic analyses identify recurrent MEF2D fusions in acute lymphoblastic leukaemia. *Nature communications* 2016 Nov 8; **7:** 13331.

5. Hormann FM, Hoogkamer AQ, Beverloo HB, Boeree A, Dingjan I, Wattel MM*, et al.* NUTM1 is a recurrent fusion gene partner in B-cell precursor acute lymphoblastic leukemia associated with increased expression of genes on chromosome band 10p12.31-12.2. *Haematologica* 2019 Oct; **104**(10)**:** e455-e459.

6. Martens JH, Stunnenberg HG. BLUEPRINT: mapping human blood cell epigenomes. *Haematologica* 2013 Oct; **98**(10)**:** 1487-1489.

7. Carrillo-de-Santa-Pau E, Juan D, Pancaldi V, Were F, Martin-Subero I, Rico D*, et al.* Automatic identification of informative regions with epigenomic changes associated to hematopoiesis. *Nucleic acids research* 2017 Sep 19; **45**(16)**:** 9244-9259.

8. Ernst J, Kellis M. ChromHMM: automating chromatin-state discovery and characterization. *Nature methods* 2012 Feb 28; **9**(3)**:** 215-216.

SUPPLEMENTARY FIGURE LEGENDS

Supplementary Figure S1. Consort diagram of Interfant-99/06 cohort screen

Karyotypes for all *KMT2A*-wildtype infant B-ALL cases (< 1 year) from the Interfant-99 and -06 cohorts were evaluated. Interfant-related study groups were approached to provide *NUTM1* screening results for cases with a karyotypic 15q aberration, a normal karyotype or missing karyotype. Cases with a known genetic subtype (*BCR-ABL1, TCF3-PBX1*, high hyperdiploidy) or other structural or numeric cytogenetic aberrations not including 15q were not specifically screened and assumed negative for *NUTM1* fusion. Cases with a 15q aberration were assumed positive for *NUTM1* fusion, which was confirmed in 13/19 cases with material available for testing.

Supplementary Figure S2. Diagram overview of study cases

Supplementary Figure S3. HOXA cluster epigenetic landscape

Comprehensive epigenetic profiles were obtained through bisulfite sequencing to detect CpG methylation of DNA (ME), RNA sequencing to detect gene expression (TPM), and chromatin immunoprecipitation sequencing (ChIP-seq) to detect histon modifications H3K4me3, H3K4me1, H3K36me3, H3K27Ac, H3K27me3 and H3K9me3 from the BLUEPRINT project.[6] The individual datasets used are listed in **Supplementary Table S10**. Chromatin segmentations were carried out with ChromHMM package (v1.10), as previously described, resulting in chromatin states (CS) E1 to E11.[7, 8] Data integration and visualization was done using R package Gviz (v1.30.3). Integrated data for five pediatric ALL cases are shown, aligning CS, ME and TPM data (y-axis) along the HOXA region on 7p (x-axis). An *ACIN1-NUTM1* fusion case (red), revealed upregulation of the HOXA gene cluster, mainly *HOXA9* and *HOXA10*, as demonstrated by increase in gene expression, decrease in DNA methylation, and active transcription in open chromatin in the *HOXA9* locus. The overall epigenetic landscape in the HOXA cluster is comparable to those observed in two cases with *KMT2A-AFF1* fusion (blue), in contrast to other ALL subtypes (black), which did not show upregulation of the HOXA gene cluster. All epigenetically profiled cases are children between 11 and 15 years old.
